# Editorial: Virus Discovery by Metagenomics: The (Im)possibilities

**DOI:** 10.3389/fmicb.2017.01710

**Published:** 2017-09-08

**Authors:** Bas E. Dutilh, Alejandro Reyes, Richard J. Hall, Katrine L. Whiteson

**Affiliations:** ^1^Theoretical Biology and Bioinformatics, Utrecht University Utrecht, Netherlands; ^2^Centre for Molecular and Biomolecular Informatics, Radboud University Medical Centre Nijmegen, Netherlands; ^3^Department of Biological Sciences, Universidad de los Andes Bogotá, Colombia; ^4^Max Planck Tandem Group in Computational Biology, Universidad de los Andes Bogotá, Colombia; ^5^Center for Genome Sciences and Systems Biology, Washington University in Saint Louis St Louis, MO, United States; ^6^Animal Health Laboratory, Ministry for Primary Industries Upper Hutt, New Zealand; ^7^Department of Molecular Biology and Biochemistry, University of California, Irvine Irvine, CA, United States

**Keywords:** metagenomics, virus discovery, virome, bacteriophages, phages, metagenome, bioinformatics, biological dark matter

This Frontiers in Virology Research Topic showcases how metagenomic and bioinformatic approaches have been combined to discover, classify and characterize novel viruses. Since the late 1800s (Lecoq, 2001), the discovery of new viruses was a gradual process. Viruses were described one by one using a suite of techniques such as (electron) microscopy and viral culture. Investigators were usually interested in a disease state within an organism, and expeditions in viral ecology were rare. The advent of metagenomics using high-throughput sequencing has revolutionized not only the rate of virus discovery, but also the nature of the discoveries. For example, the viral ecology and etiology of many human diseases are being characterized, non-pathogenic viral commensals are ubiquitous, and the description of environmental viromes is making progress.

This accelerated rate of virus discovery comes both with fantastic possibilities and with significant risks. Metagenomics has already unveiled vast microbial biodiversity in a range of environments, and is increasingly being applied in clinics for difficult-to-diagnose cases. Hall et al. have contributed a thoughtful review on the challenges in using viral metagenomics for diagnostics, including handling of incidental findings, implications for agricultural and horticultural trade, privacy concerns relating to the host's genome, data sharing, cost, quality assurance, and etiology. Presently, the genomic era defines the viral universe by characterizing genotypes, but these genotypes are rarely associated with a phenotype and/or a physical entity. Moreover, the number and diversity of viral sequences in reference databases are dwarfed by the sequences from their cellular hosts. As a result, state of the art taxonomic classification of viruses recognizes only several thousand viral species, a large fraction of which infect humans. This stands in sharp contrast with the diversity of the cellular organisms on which all viruses depend for their replication.

Although fifteen years have passed since the first viral metagenome was sequenced from an ocean sample (Breitbart et al., 2002), the experimental and bioinformatic methods used for viral metagenomics have not reached a consensus. First, this reflects the diversity in applications ranging from virus discovery, to diagnostics, to ecological surveys. Second, this reflects the diversity in the microbial world itself that includes giant viruses (Halary et al., 2016), tiny bacteria (Brown et al., 2015), and everything in between. Thus, even the most basic experimental steps such as filtering viruses from an environmental sample need to be optimized for different applications. Viruses may employ diverse genomic molecular compositions, as illustrated by an RNA sequencing study that uncovered several single stranded and double-stranded RNA viruses in mosquitoes (Chandler et al.). Roux and colleagues studied the uncultivated “Far-T4 phages” that are commonly found in aquatic environments, identifying five clades with largely collinear genome organizations (Roux et al.). Our Research Topic highlights the diversity of the virosphere in reviews on plant viruses where metagenomics has revealed an unexpected diversity in viruses with persistent lifestyles (Roossinck), and on coral reefs where herpes viruses were revealed in various host species (Houldcroft and Breuer).

A third reason for the diversity in viral metagenomics methods is the ongoing development of new tools and approaches, that are both cause and effect of our improved understanding of the virosphere. Bioinformatically, identifying viral sequences in a shotgun metagenomic dataset can be like finding a needle in a haystack (Soueidan et al.). Detection methods based on reference sequences can sensitively identify known viruses in short-read datasets (Pirovano et al.), but may limit the search to identify only known species. A promising possibility is to use short seeds to identify and progressively assemble viral sequences from the dataset, for example allowing the reconstruction of 45 partial or complete *Alpavirinae* genomes (Alves et al.). Alternatively, abundance and nucleotide usage signals can be used to identify *de novo* assembled metagenomic contigs belonging to the same genome, although the specificity of these binning signals varies (Smits et al.). Viral fosmids are a complementary approach allowing the recovery of long contiguous sequences, albeit at the cost of an inherently lower throughput. Chow et al. combined fosmid sequencing with shotgun metagenomics and database searches to chart the viral diversity in a Canadian fjord, elucidating genomic and ecological contexts, and identifying potential host interactions.

It has been known for decades that viruses infect economically important crops and animals including humans. However, a different view of viruses has recently emerged, the virome, a name for the entire community of viruses found in a given biome. For example, the human virome consists of the viruses that normally live on and within a human being, and includes viruses that infect the human itself, but also viruses ingested via food and the viruses infecting human-associated bacteria and archaea. Indeed, viruses are everywhere, and understanding the role of the virome within a complex ecosystem is a challenge at a whole new level. Aziz et al. developed various metrics to represent the presence of sequences in metagenomes and environments, and created a web tool showing the presence of a set of reference genomes in available metagenomes.

In our Research Topic, different approaches were taken. The bacteriophage adherence to mucus (BAM) model, which proposed that bacteriophages could assist the immune system of animal hosts by creating an external layer of defense in mucosal surfaces (Barr et al., 2013), has been extended to wild and farmed eels (Carda-Dieguez et al.). While the BAM model suggests that mainly lytic phages should benefit from this behavior, very similar Ig-like motifs to those originally implicated in mucus attachment were identified in temperate *Pseudomonas aeruginosa* phages (Tariq et al.). Those findings led to a proposed model incorporating BAM into the lifecycle of *P. aeruginosa* in cystic fibrosis patients.

Some viruses in host-associated viromes may chronically linger without causing any symptoms or phenotype, until their emergence is triggered, for example following debilitation of the host immune system in the case of eukaryotic viruses, or other environmental stresses in the case of temperate bacteriophages progressing into the lytic cycle. For example, Santiago-Rodriguez et al. identified a phage in Methicillin Resistant *Staphylococcus aureus* (MRSA) whose expression was inhibited in an *ex vivo* human blood model, suggesting preference for the lysogenic state in blood. The ecological question of virome stability is also relevant outside the context of a host, being linked to organismal diversity and nutrient release (Suttle, 2007). In our Research Topic, marker gene amplification studies targeting *Picornavirales* (Gustavsen et al.) and *Gokushoviruses* (Labonté et al.) in Canadian coastal waters show high viral diversity, both spatiotemporally and across a depth gradient. This variability suggests an important role for the viruses in structuring the bacterial and eukaryotic plankton community, as well as in nutrient cycling and energy transfer.

Santiago-Rodriguez et al. investigated whether the virome could be used as a sensitive marker of alterations in the health status of a host. While the urinary tract was long considered a sterile environment except during rare urinary tract infections (UTIs), it was recently shown that in fact, it contains an associated microbiome even in healthy individuals. Like many human-associated viromes, the urinary tract virome sequences were found to be dominated by bacteriophages. Only 27% of virome contigs were homologous to a known virus (similar to what other studies of human viromes find), and most of the hits matched bacteriophages. No significant changes were detected between healthy individuals and UTI patients (Santiago-Rodriguez et al.). Interestingly, human papillomavirus (HPV) was detected in 95% of the subjects, regardless of disease status. Traditionally, HPV was associated with diseases including cancer, but many HPV genotypes are now widely detected without any apparent association to disease. A similar case is a novel gamma-papillomavirus that was discovered in the virome of a patient with a respiratory tract infection (Canuti et al.). This HPV was present at equivalent titers during the respiratory infection and after the recovery, suggesting it was not the cause of the disease.

Linking unknown viral metagenomic sequencing reads to a function in a complex environment is often impossible, so in vitro systems to study novel phage bacteria interaction could serve as an ideal intermediate. For example, Gambelli et al. characterized the virome in a bioreactor containing a *Methylomirabilis oxyfera* enrichment culture, hoping to identify a phage that infected this important nitrogen cycling bacterium. While the shape and size of the virions could be modeled in high detail by advanced electron microscopy and three-dimensional imaging, and high-throughput metagenomic sequencing identified several very long bacteriophage contigs, it still proved challenging to identify which of the metagenomic sequences represented the phages the *M. oxyfera* phages seen in the images (Gambelli et al.). This impressive effort thus highlights some of the challenges we face on the road ahead toward a full understanding of viruses and their interactions in the natural environment.

## Conclusions and outlook

Studying viral sequences means working at the edge of human knowledge. Even microbial genomics experts working on uncultivated microbes use the term “dark matter” when describing the viral sequences they find in metagenomes. While metagenomics expands our ability to detect viruses, a combination of small viral sequence databases and great diversity still means that many viral reads have no homology to known viruses (Mokili et al., 2012). Whether they are host-associated or free-living, we now know that most viruses (like microbes in general) are not pathogenic to humans, plants, or animals. Recent technological advances including decreased DNA sequencing costs and the development of novel methods in metagenome analysis are making the study of viral communities feasible to many laboratories around the world. The Research Topic authors were motivated to identify novel viral agents of disease, illuminate the vast “dark matter” that is viral diversity, discover functional genes carried by bacteriophages, uncover how phages structure microbial communities, and perhaps support renewed interest in phage therapy to target antibiotics resistant bacterial infections. The current Research Topic is an excellent compendium of manuscripts that, far from being comprehensive, we hope will form a foundation and inspiration for many other studies to come in the field of viral discovery, and motivate a new generation of microbial ecologists to include the viruses in their research.

## Author contributions

BD, AR, RH, and KW edited the Research Topic and wrote the Editorial.

### Conflict of interest statement

The authors declare that the research was conducted in the absence of any commercial or financial relationships that could be construed as a potential conflict of interest.
